# Solid-State Polymerization as a Vitrimerization Tool Starting from Available Thermoplastics: The Effect of Reaction Temperature

**DOI:** 10.3390/ma14010009

**Published:** 2020-12-22

**Authors:** Christos Panagiotopoulos, Athanasios Porfyris, Dimitrios Korres, Stamatina Vouyiouka

**Affiliations:** Laboratory of Polymer Technology, School of Chemical Engineering, Zographou Campus, National Technical University of Athens, 157 80 Athens, Greece; chpanagiotopoulos@mail.ntua.gr (C.P.); adporfyris@mail.ntua.gr (A.P.); dmkorres@central.ntua.gr (D.K.)

**Keywords:** poly(butylene terephthalate), glycerol, vitrimers, solid state polymerization, crosslinking, crystallization, reprocessability, dynamic covalent adaptable network

## Abstract

In the current work, solid-state polymerization (SSP) was studied for the synthesis of poly(butylene terephthalate), PBT-based vitrimers. A two-step process was followed; the first step involved alcoholysis reactions and the incorporation of glycerol in the polymer chains. The second step comprised transesterification reactions in the solid state (SSP) in the presence of zinc(II) catalyst resulting in the formation of a dynamic crosslinked network with glycerol moieties serving as the crosslinkers. The optimum SSP conditions were found to be 3 h at 180 °C under N_2_ flow (0.5 L/min) to reach high vitrimer insolubility (up to 75%) and melt strength (2.1 times reduction in the melt flow rate) while increasing the crosslinker concentration (from 3.5 to 7 wt.%) improved further the properties. Glass transition temperature (*T_g_*) was almost tripled in vitrimers compared to initial thermoplastic, reaching a maximum of 97 °C, whereas the melting point (*T_m_*) was slightly decreased, due to loss of symmetry perfection under the influence of the crosslinks. Moreover, the effect of the dynamic crosslinked structure on PBT crystallization behavior was investigated in detail by studying the kinetics of non-isothermal crystallization. The calculated effective activation energy using the Kissinger model and the nucleating activity revealed that the higher crosslinker content impeded and slowed down vitrimers melt crystallization, also inducing an alteration in the crystallization mechanism towards sporadic heterogeneous growth.

## 1. Introduction

For many years, polymers have been divided into two main categories according to their behavior upon heating: thermoplastics and thermosets. Thermoplastics can be melted and recycled, easily and quickly processed using techniques such as extrusion or injection, but present inferior thermal and mechanical properties, especially at high temperatures and solvents. In contrast, thermosets exhibit excellent mechanical properties, durability and chemical stability, which render them ideal for multiple challenging applications, such as in the aircraft and automobile industries. Nonetheless, due to the development of a stable and dense chemical network, thermosets cannot flow or dissolve once synthesized and thus, they cannot be recycled, reprocessed or self-healed [1,2,3,4,5,6]. The challenge is to bridge the gap between classical thermosets and thermoplastic polymers and overcome limitations of conventional materials regarding lifetime, safety and environmental impact [7].

Vitrimers constitute a new class of covalent adaptable networks (CANs), in which thermally stimulated associative exchange reactions to allow the topological rearrangement of the dynamic network while keeping the number of the bonds and crosslink density constant [1,2,4,6]. Pioneered by Leibler and coworkers [3,8], epoxy-based crosslinked structures offered the unique ability to reprocess/recycle under heating: these vitrimers belong to a transesterification-based category and exhibit attractive characteristics due to the high availability of monomers and ease of synthesis, which render them readily up-scalable and applicable in an industrial context. They are synthesized upon heating mixtures of bi- and poly-functional monomers in the presence of a proper catalyst. Indicatively, in the initial works of Leibler et al. [3,8], epoxy-based vitrimers were prepared using diglycidyl ether of bisphenol A (DGEBA), a mixture of fatty dicarboxylic and tricarboxylic acids (1:1 epoxy/COOH stoichiometry) in the presence of 5 to 10 mol % zinc(II) acetylacetonate hydrate (Zn(acac)_2_) at 150 °C. The hard epoxy-resin material obtained could rearrange its topology upon heating by associative exchange reactions, i.e., at 200 °C without depolymerization demonstrating insolubility and reprocessing capability.

However, the challenge is to use metal-epoxy chemistry to synthesize semi-crystalline vitrimers from commercial thermoplastics by a crosslinking method that can be applied on an industrial scale. In this direction, vitrimers have been developed in a number of studies based on poly(lactic acid) (PLA) [1], high-density polyethylene (HDPE) [9,10], poly(methyl methacrylate) (PMMA) [10,11], polystyrene (PS) [10] and poly(butylene terephthalate) (PBT) [12,13,14,15]. PBT is proved suitable for a wide range of industrial applications but its low number-average molecular weight ($\bar{M_{n}}$ 5000 to 45,000 g/mol) and melt strength result in difficulties in melt processing and in high-temperature applications, e.g., electronic applications [12,16,17,18,19,20]. PBT-based vitrimers were designed [12] through reactive extrusion by adding DGEBA (0–4.2 wt.%), esterification catalyst (2-methylimidazole, 2-MI) or zinc(II) transesterification catalyst (Zn(acac)_2_) in a mini compounder for 10 min at 270 °C to increase PBT melt strength. No gelation was achieved with the use of 2-ΜΙ, while Zn(acac)_2_ efficiently catalyzed the crosslinking, providing vitrimers with insolubility up to 75% and generally similar thermal properties compared to neat PBT (*T_g_* ~48 °C, *T_c_* ~170 °C and *T_m_* ~210 °C). 

Nevertheless, reactive extrusion is not suitable for vitrimers formation in the case of volatile, thermal sensitive comonomers, such as polyols, along with the fact that thermomechanical degradation of the polymer may occur [12]. Crystallinity and mechanical properties are also affected in the melt due to the randomization of the chemical structure, crystal packing and crosslinking. These drawbacks of vitrimers melt-based synthesis can be prevented by using a solid-state polymerization (SSP) process. SSP constitutes a bulk polymerization technique, applied on an industrial scale for polycondensates. SSP involves heating the prepolymer to a temperature between the glass transition point (*T_g_*) and its melting temperature (*T_m_*) [21,22]. SSP has already been used to chemically modify the PBT backbone in the works of Koning et al. [16,19,23,24], where bicyclic carbohydrate-based diols (2,3:4,5-di-*O*-methylene-galactitol (Galx) and 2,4:3,5-di-*O*-methylene-*D*-mannitol (Manx)) or bio-based fatty acid dimer diol (FADD) were introduced into the macromolecular chain. These endeavors resulted in copolyesters with a unique block-like chemical microstructure that provided superior thermal properties compared to the random counterparts obtained by melt copolymerization. In this direction, PBT-based vitrimers via SSP were studied by Zhou et al. [13,14,15], in which the effect of glycerol concentration (2 to 18% mol) and SSP residence time (1 to 7 h) on vitrimers thermomechanical and rheological properties were examined. Tunable rheological behavior ranging from a typical thermoplastic to a solid-like gel was achieved by controlling the exchange dynamics and crosslinks density mainly via variations in the catalyst to crosslinker molar ratio.

In the current work, the advance lies in developing a two-step SSP technique as a crosslinking tool to synthesize PBT-based vitrimers with PBT-like characteristics and enhanced melt strength while investigating the effect of reaction temperature upon the crosslinking extent, the rheological behavior, and the thermal characteristics. In particular, the main objective herein was to investigate the influence of the crosslinker (glycerol) concentration on the alcoholysis reactions occurring at the first step of the process as well as to establish a correlation between the most crucial repolymerization parameters (reaction temperature, glycerol content) and the end products properties, such as insolubility, melt strength and thermal transitions. Even more, the melt crystallization of vitrimers was investigated for the first time to the best of our knowledge, as crystallization kinetics is a key factor affecting the physical and mechanical properties of the end-product during the subsequent melt processing and mechanical recycling. The establishment of an appropriate vitrimerization route via SSP for thermoplastic-based vitrimers is anticipated to provide a practical, economically sustainable solution that will enhance their performance and strengthen their application as engineering polymers. 

## 2. Materials and Methods

**Materials.** Poly(butylene terephthalate) (≥99%) in pellets (PBT) (Enplast Plastik, Istanbul, Turkey) was cryomilled under liquid nitrogen into powder using a pulverisette grinding mill (Fritsch GmbH, Idar-Oberstein, Germany). PBT powder was then dried at 120 °C for 6 h under vacuum. Glycerol (≥99.5%), Zinc(II) acetylacetonate hydrate (Zn(acac)_2_) in powder (≥99%), acetone (≥99.5%) (Sigma-Aldrich, Saint Louis, MO, USA) and 1,1,1,3,3,3-Hexafluoro-2-propanol (HFiP) (≥99%) (Merck Schuchardt OHG, Hohenbrunn, Germany) were used as received.

**Preparation of PBT/glycerol/zinc(II) catalyst physical mixtures.** Two distinct physical mixtures of PBT, glycerol and Zn(acac)_2_, were prepared from solution at two different concentrations of glycerol, referred to as G3.5 and G7, in respect to PBT. For the G3.5 mixture (3.5 wt.% glycerol), 9.14 g dried PBT powder, 0.32 g glycerol, and 0.22 g Zn(acac)_2_ were precisely weighed, while for the G7 mixture (7 wt.% glycerol), 0.64 g of glycerol were used. Each mixture was added in a 100 mL round-bottom flask along with 20 mL of HFiP at 55 °C in order to dissolve the solids, under gentle magnetic stirring with reflux. After the solids were completely dissolved, the solvent was recovered in a Buchi Rotavapor R-210 rotary evaporator (Buchi Corp., New Castle, DE, USA) under vacuum; the solid residue was dried under vacuum for 24 h at 30 °C and cryomilled into powder, using a Fritsch Pulverisette grinding mill. The powder was collected and dried again under vacuum for 24 h at 30 °C.

**Alcoholysis/incorporation of glycerol into PBT—first step.** PBT-based vitrimers were prepared via a two-step process. In the first step, the alcoholysis of the PBT/Glycerol/Zn(II) physical mixtures took place: typically, 5 g of each physical mixture (G3.5 and G7) were added into a cylindrical stainless steel fixed-bed reactor, pressurized with N_2_ (P < 3 bar) in order to avoid glycerol evaporation and left for 24 h at 160 °C to incorporate the glycerol molecules into the PBT chains. Neat pulverized PBT was also subjected to the same procedure for comparison reasons.

**Solid-state polymerization/crosslinking step—second step.** The alcoholysis products from the 1st step were subjected to SSP under continuous N_2_ flow (0.5 L/min) at three different repolymerization temperatures (180 °C, 190 °C and 200 °C) in a stainless steel fixed-bed reactor. The reactor was purged several times with N_2_ prior to the SSP, and when the temperature inside the reactor reached the desired value, the measurement of the reaction time *t_ssp_* initiated. At the end of the SSP (*t_ssp_* = 3 h), the reactor was gradually cooled down to room temperature under constant N_2_ flow, and the final vitrimer product was dried under vacuum for 6 h at 120 °C. Similarly, the neat alcoholyzed PBT powder was subjected to SSP for comparison reasons.

**Characterization.***Dilute solution viscometry***.** Viscosity measurements were performed in the neat PBT powder, physical mixtures of PBT/Glycerol/Zn(II) and the alcoholysis products (1st step). Samples were dissolved in HFiP (0.5% w/v) at room temperature and filtered prior to measurement using a disposable membrane. Measurements were performed employing an Ubbelohde capillary viscometer at 25 ± 0.1 °C, and the intrinsic viscosity ([*η*], dL/g) was determined using Equation (1):

(1) $$\left[\eta \right]=\frac{\sqrt{1+1.5\left(\frac{t}{t_{o}}-1\right)}-1}{0.75C}$$

where *t* (s) is the flow time of solution, *t_o_* (s) is the flow time of pure solvent and *C* (g/100 mL of solvent) is the solution’s concentration normalized to the polymer weight in the physical mixtures.

Subsequently, the intrinsic viscosity was converted to $\bar{M_{n}}$ via Equation (2) [25]:

(2) $$\left[\eta \right]=2.31\;\cdot \;10^{-4}{\bar{{Μ}_{n}}}^{0.80}$$

*Insolubility test.* Vitrimers insolubility was measured with dissolution in HFiP and calculation of gel fraction (*G_f_*, %) (Equation (3)). In particular, ca. 50 mg (*m_initial_*) of vitrimer were added in 5 mL of HFiP and left for 24 h to dissolve at room temperature. The insoluble residue was isolated by filtration, dried for 6 h at 120 °C under vacuum and finally weighed accurately (*m_final_*).

(3) $$G_{f}\;\left(\%\right)=\frac{m_{final}}{m_{initial}}\;\cdot \;100\%$$

*Melt flow rate (MFR).* The melt flow rate (MFR, g/10 min) was measured at 250 °C under 2.160 kg, according to the ASTM-D1238 standard, using a Dynisco model 4004 capillary rheometer (Dynisco LLC, Franklin, MA, USA). The MFR was expressed in grams of polymer per 10 min, according to Equation (4):

(4) $$MFR\;\left(250,\;2.16\right)=\frac{600}{t}\;\cdot \;m_{avg}$$

where *t* (s) is the time between consecutive cuts of the molten material (cut-off interval was 10 sec) and *m_avg_* (g) is the average mass of the material extruded.

*Thermal properties—melt crystallization kinetics.* Differential scanning calorimetry (DSC) measurements were performed in a Mettler DSC 1 STARe System (Mettler-Toledo International Inc, Columbus, OH, USA), through a heating-cooling-heating cycle from 30 up to 280 °C under N_2_ flow (20 mL/min) using heating-cooling rates at 10 °C/min. The melting points (*T_m_*, °C) were derived from the first and second heating cycles, hereby represented as *T_m_*_1_ and *T_m_*_2_, and the relevant mass fraction crystallinity (*x_c_*, %) represented as *x_c_*_1_ and *x_c_*_2_, were computed according to Equation (5):

(5) $$x_{c}=\frac{\Delta {Η}_{m}}{\Delta {Η}_{0}}\;\cdot \;100\;\%$$

where *x_c_* (%) is the degree of crystallinity, Δ*H_m_* (J/g) is the melting enthalpy, and Δ*H*_0_ (J/g) is the melting enthalpy of 100% crystalline PBT that equals to 145 J/g [26].

The mobile amorphous fraction, hereby referred as *a_mobile_* (%), was calculated using the heat capacity increase at half-step Δ*C_p_* from the glass transition (*T_g_*, °C) obtained during the first heating cycle and, using Equation (6):

(6) $$a_{mobile}=\frac{\Delta C_{p}}{\Delta C_{p}^{a}}\;\cdot \;100\;\%$$

where *a_mobile_* (%) is the mobile amorphous fraction, Δ*C_p_* (J/g∙K) is the heat capacity increase at half step and $\Delta C_{p}^{a}$ (J/g∙K) is the heat capacity increase at the half step of 100% amorphous PBT that equals to 0.35 J/g∙K [26].

Subsequently, the rigid amorphous fraction, hereby referred as *a_rigid_* (%), was calculated using Equation (7):

(7) $$a_{rigid}=100\;\%-a_{mobile}-x_{c}$$

where *a_rigid_* (%) is the rigid amorphous fraction, *a_mobile_* (%) is the mobile amorphous fraction, and *x_c_* (%) is the degree of crystallinity.

The crystallization point (*T_c_*, °C) as well as the crystallization enthalpy (Δ*Η_c_*, J/g) were obtained from the DSC cooling cycle. Regarding the melt crystallization study, the relative degree of crystallinity was calculated as a function of temperature *X(T)* using Equation (8):

(8) $$X\left(T\right)=\frac{\int_{T_{0}}^{T_{c}}\left(\frac{dH_{c}}{dT}\right)dT}{\int_{T_{0}}^{T_{\infty }}\left(\frac{dH_{c}}{dT}\right)dT}$$

where *dH_c_* (J/g) is the measured enthalpy of crystallization during an infinitesimal time interval dt, *T*_0_ (°C) is the initial crystallization temperature, *T_c_* (°C) is the crystallization temperature as a function of time *t*, and *T_∞_* (°C) is the temperature after the completion of the crystallization process.

The crystallization temperature *T_c_* was correlated to the crystallization time *t* using Equation (9) [27,28]:

(9) $$t=\frac{T_{0}-T_{C}}{a}$$

where *a* (°C/min) is the constant cooling rate.

Thermogravimetric analysis (TGA) was also conducted in a Mettler TGA/DSC 1 thermobalance (Mettler-Toledo International Inc, Columbus, OH, USA) using a heating rate of 10 °C/min from 30 to 800 °C under constant N_2_ flow (20 mL/min). The onset decomposition temperature (*T_d,_*_5*%*_, °C) was defined as the temperature at 5% weight loss, the degradation temperature (*T_d_*, °C) was determined at the maximum rate of weight loss and the char yield as the percentage residue (*W*, %) at 800 °C. The materials produced, the neat PBT powder, glycerol and the catalyst were accordingly analyzed.

## 3. Results and Discussion

**Alcoholysis—incorporation of glycerol into PBT**

In the current study, neat pulverized PBT (*t* = 0) was the reference material exhibiting a relatively low $\bar{M_{n}}$ (29,000 g/mol) and a high MFR value (29.3 g/10 min) (Table 1), which underlines its low melt strength and the difficulty to be used in processes involving elongated flows such as extrusion, film blowing, foaming etc. [13,19]. The initial molecular weight of PBT was comparable to the one studied by Zhou et al. [13] (21,200 g/mol), whereas PBT was intended for reactive extrusion studied by Demongeot et al. [12] presented a 1.6 times higher molecular weight (47,000 g/mol). Conveniently, the low molecular weight of PBT allowed us to introduce a significant amount of glycerol (3.5 and 7 wt.%) via polymer easier solubilization compared to a higher molecular weight grade. Regarding thermal properties (Table 2), the pulverized PBT was found semi-crystalline with *x_c_* 34–35% and thermally stable up to 361 °C, while the degradation temperature peaked at 391 °C and the residue was 6%, in accordance with similar PBT studies [18].

After cryomilling, the PBT/glycerol/Zn(II) physical mixtures were free-flowing in the form of white powder, also demonstrating an apparent increase in the solution intrinsic viscosity values (Table 1). At the same time, a lower MFR was recorded, 24.2 and 21.7 g/10 min for the G3.5 and G7, respectively. These diffusion-related changes can be attributed to the inflexibility and bulkiness of glycerol and catalyst in the physical mixtures, which increased the flow time both in solution and melt. For the same reasons, the physical mixtures demonstrated considerable alterations in their thermal properties (Table 2) compared to neat PBT powder. Specifically, *T_g_* greatly increased from 35 °C to 84 °C and 89 °C for G3.5 and G7, respectively, implying that glycerol and catalyst molecules were accommodated in the amorphous regions of the polymer. On the other hand, *x_c_* and *T_m_* were decreased at a much lower extent (compared to *T_g_*) for both mixtures due to the incorporation of chain imperfections into the crystal lattice. In terms of TGA analysis, the physical mixtures G3.5 and G7 did not exhibit noteworthy differentiation in the *T_d,_*_5*%*_ (360–361 °C) and *T_d_* (398 °C), while the residue was found slightly higher (7.9–8.1%) due to the different composition (catalyst, glycerol) compared to neat PBT (6.3%).

For comparison reasons, the neat PBT powder was also subjected to the first step of the process (alcoholysis, 24 h at 160 °C). Absent the crosslinker and catalyst; the polymer properties remained basically constant: the relevant change in the $\bar{M_{n}}$ was insignificant, from 29,000 to 28,000 g/mol, and MFR increased slightly (29.3 to 32.1 g/10 min), obviously due to restricted chain scission reactions [29]. Similarly, small variations were evidenced in the neat PBT thermal properties: it maintained its *T_g_* (31–33 °C) and *x_c_* (30–34%) while the *T_m_* (224–225 °C) tended to decrease for both heating cycles. The degradation characteristics were also maintained at the same levels, recording *T_d,_*_5*%*_ ~361 °C, *T_d_* ~391 °C and residue at 6%.

**Figure 1:**
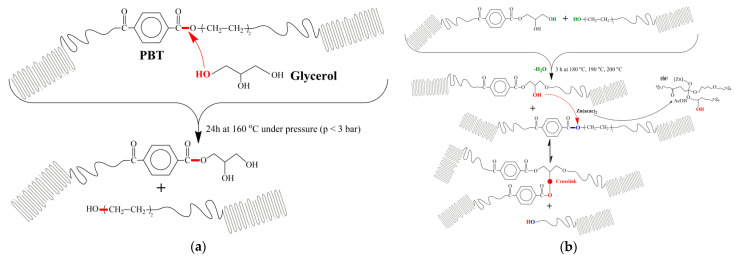
(**a**) Alcoholysis reactions of PBT in the presence of glycerol, (**b**) transesterification reactions of PBT with glycerol in the presence of Zn(acac)_2_ during the solid state polymerization step of the process.

During the alcoholysis step of the physical mixtures, glycerol is anticipated to be incorporated into the PBT chains via random chain scission of ester linkages (red links), as depicted in Figure 1a. The alcoholyzed products were obtained as free-flowing, colorless powders with no sign of crosslinked fractions as they were readily soluble in HFiP. They presented significantly lower [*η*] values (Table 1) due to the alcoholysis reactions, and the relevant decrease was found proportional to the glycerol concentration, i.e., ca. 55% reduction in $\bar{M_{n}}$ for the G3.5 and 66% for the G7. In fact, the herein recorded molecular weight after the alcoholysis step (24 h at 160 °C) was found strikingly higher compared to the study of Zhou et al. [13] for analogous crosslinker concentration and reaction conditions, i.e., 84% $\bar{M_{n}}$ reduction for 7 wt. % glycerol. This milder $\bar{M_{n}}$ decrease of our alcoholyzed products is evidently promising since the polymer was practically not extensively deteriorated by the alcoholysis reactions; we managed just to diminish the molecular weight so as to successfully incorporate the glycerol molecules. 

MFR measurements also confirmed the occurrence of alcoholysis reactions: it was notably increased up to 59.6 and 66.0 g/10 min for G3.5 and G7, respectively, ascribed to the chain scission by the free glycerol hydroxyl groups. DSC analysis was further employed: as expected, the alcoholyzed products presented inferior thermal properties, starting with the *T_g_*_1_, which decreased to 50 °C (G3.5) and 46 °C (G7), being relatively closer to the neat polymer. During this initial step of the process, two mechanisms can cumulatively affect the *T_g_*; incorporated glycerol may act as a plasticizer increasing the molecular mobility of the polymer chains, while simultaneously the large $\bar{M_{n}}$ decrease progressively augmented the free volume enabling the smaller chains to diffuse and flow even more easily. Unavoidably, the crystalline regions were also affected during the alcoholysis step, and as a result, *x_c_* and *T_m_* of alcoholyzed products were decreased for both heating cycles compared to the neat PBT powder (Table 2). Finally, the alcoholyzed products similarly underwent a one-stage mass loss in TGA and generally maintained the same thermal stability as the pre-alcoholysis physical mixtures G3.5 and G7.

In order to verify that glycerol was solely incorporated into the polymer’s amorphous regions by alcoholyzing the polymer chains, the compound’s morphology was monitored before and after this step by further processing the DSC data. PBT, as a semi-crystalline thermoplastic polymer, demonstrates a three-phase morphology as firstly proposed by Jansen et al. [16,24], in which a small fraction of the amorphous phase is confined by the crystalline chain segments. Therefore, the amorphous regions were subdivided into *a_mobile_* and *a_rigid_*, which were calculated via Equations (6) and (7) (Table 2). Initially, the neat PBT and physical mixtures G3.5 and G7 possessed a significant fraction of *a_rigid_*: 43.6% for PBT, 57.0 and 62.8% for G3.5 and G7, respectively, and a lower fraction of *a_mobile_*: 21.1% for PBT, 10.8 and 6.6% for G3.5 and G7, respectively, due to the decreased segmental mobility of the polymer chains and the rigidity of the materials, as previously discussed for the *T_g_* increase. Then, with the alcoholysis step, an important conversion of *a_rigid_* into *a_mobile_* was observed, especially in the case of the G7 compound (*a_mobile_* increased from 6.6 up to 55.9%) due to chain scission reactions, which implies that glycerol was completely incorporated into PBT. On the contrary, neat PBT practically maintained the same values of rigid and mobile amorphous regions, further emphasizing that in the absence of crosslinker and catalyst, there were no alcoholysis reactions (Table 2). These findings of our work do not agree with the results of Zhou et al. [13], who reported no significant modification of *a_rigid_* and *a_mobile_* during the alcoholysis step; the authors rather suggested that after this first step, the material still exclusively consisted of rigid amorphous and crystalline fractions.

**Solid-state polymerization/crosslinking step of the process**

The alcoholyzed neat PBT and physical mixtures G3.5 and G7 were subjected to solid state repolymerization in which transesterification reactions between end groups of the chains are anticipated to occur in the presence of Zn(acac)_2_ (Figure 1b). Three different SSP temperatures were examined (180, 190 and 200 °C) for 3 h, operating at ca. 2 °C–35 °C below the alcoholyzed products *T_m_*. The relevant temperatures permitted the maintenance of the solid-state character of the process since no sintering phenomena were observed except for the G7 vitrimer produced at 190 °C ((*T_m_* – *T_SSP_*) = 11 °C) and 200 °C ((*T_m_* – *T_SSP_*) = 2 °C). This phenomenon can be associated with the chain scission in the alcoholysis step leading to lower melting regions. It becomes evident that by increasing the SSP temperature and the crosslinker content, we were approaching the crystals’ melting point, where these sintering phenomena were more likely to occur. Furthermore, at the same high SSP temperatures, both G3.5 and G7 vitrimers exhibited a mild yellowish color, ascribed to a small extent thermal degradation (especially of the catalyst), while the rest of the samples maintained their white, colorless powder form. Regarding the pure PBT powder at the end of SSP, its rheological and thermal properties did not alter crucially, and it was still found soluble in HFiP. However, at the higher temperatures of 190 °C and 200 °C, the properties tended to reduce (Table 3), as the material was thermally stressed, and chain scission reactions dominated over any repolymerization during SSP.

**Figure 2:**
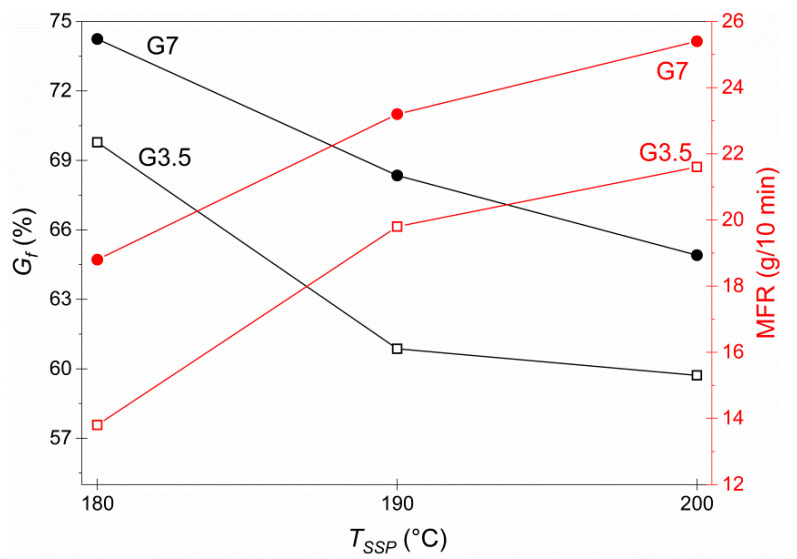
Highest crosslinking extent (*G_f_*) and highest melt strength (MFR) of the produced vitrimers G3.5 and G7 as a function of solid-state polymerization (SSP) temperature (180 °C, 190 °C and 200 °C) after 3 h of reaction.

On the other hand, the alcoholyzed physical mixtures were efficiently crosslinked after 3 h at the selected solid-state repolymerization temperatures (180 °C, 190 °C and 200 °C). SSP products presented insolubility in HFiP, which was further quantified based on the calculated gel fractions (Figure 2). As anticipated, there were amorphous and crystalline regions inside the material that did not participate in the crosslinking and thus, dissolved partially in HFiP, recording *G_f_* from 60 to 75% approximately. The increase in the glycerol concentration had a positive effect on the network development due to the higher segmental mobility as evidenced by the previously high *a_mobile_* of the alcoholyzed G7 product (Table 2) and thus higher repolymerization rate, leading to more extensive crosslinking in G7 vitrimer. This finding was also verified by a sharp decrease of G7 MFR at each temperature compared to G3.5. Indicatively at 180 °C with the highest *G_f_*, MFR was 18.8 g/10 min for G3.5 and 13.8 g/10 min for G7 (Figure 2). This observation clearly states that the crosslinking extent of PBT vitrimers is primarily affected by the glycerol content, while the feasibility of MFR measurements in SSP products proved the dynamic character of the crosslinks: at the temperature of MFR tests (250 °C), the exchange reactions enabled the movement of the crosslinks through the network, rendering the vitrimers malleable while exhibiting a high *G_f_* at room temperature. Generally, the high crosslinking extent and the enhanced melt strength of the obtained vitrimers implied that no significant amount of unreacted glycerol remained in the mixture, which would negatively affect their properties.

Examining the effect of SSP transesterification temperature on vitrimer properties, 180 °C can be considered as the optimum *T_SSP_*, in which crosslinking occurred at the highest extent, recording a maximum *G_f_* for both vitrimers G3.5 (69.8%) and G7 (74.2%) combined with the lowest MFR (18.8 and 13.8 g/10 min, respectively). Temperatures less than 180 °C were considered insufficient to undergo SSP, as the system lacks the energy and chain mobility necessary for the crosslinking formation. This is clearly supported by the fact that during the alcoholysis step of the process at 160 °C, no crosslinked structure was observed since the alcoholyzed products were still soluble to HFiP as previously underlined. On the contrary, the elevated temperatures (190 °C and 200 °C) favored the thermal decomposition of the polymer chains and limited the SSP effectiveness in terms of transesterification rate. Moreover, probably, the catalyst’s thermal degradation was also accelerated (catalyst *T_d_* = 210 °C). These findings underline that in elevated temperatures, vitrimerization is hindered by side reactions and/or thermal degradation of the compounds. Therefore, it is safe to argue that SSP is the most promising way towards vitrimerization, rather than reactive extrusion, which generally operates at much higher temperatures. Evidently, Demongeot et al. [12] observed that during reactive extrusion for an epoxy-resin system at 270 °C for 10 min, the accelerated degradation of PBT resulted in olefinic end group creation side reactions (esterification, etherification) and rearrangements through THF elimination, which further impeded vitrimerization. 

The DSC thermograms were analyzed by using the first heating run in order to acquire information on the thermal properties of the vitrimers directly after SSP. Apart from chemical resistance and malleability, the rigidity and molecular weight are anticipated to be enhanced with vitrimerization, while the free volume between the polymer chains to be diminished due to the dense crosslinking. Indeed, the SSP products exhibited an increased *T_g_* reaching a maximum of 92 °C and 97 °C for G3.5 and G7 vitrimers, respectively, almost tripled compared to neat PBT powder. Similarly, in the work of Zhou [13,15], *T_g_* increased after vitrimerization with increasing glycerol content, indicatively from 55 °C (pure PBT) up to 68 and 82 °C for 2.5 and 12 mol % glycerol, respectively. Interestingly, our vitrimers exhibited a sharper *T_g_* increase, which can be ascribed to a denser and more rigid crosslinked network. 

**Figure A1:**
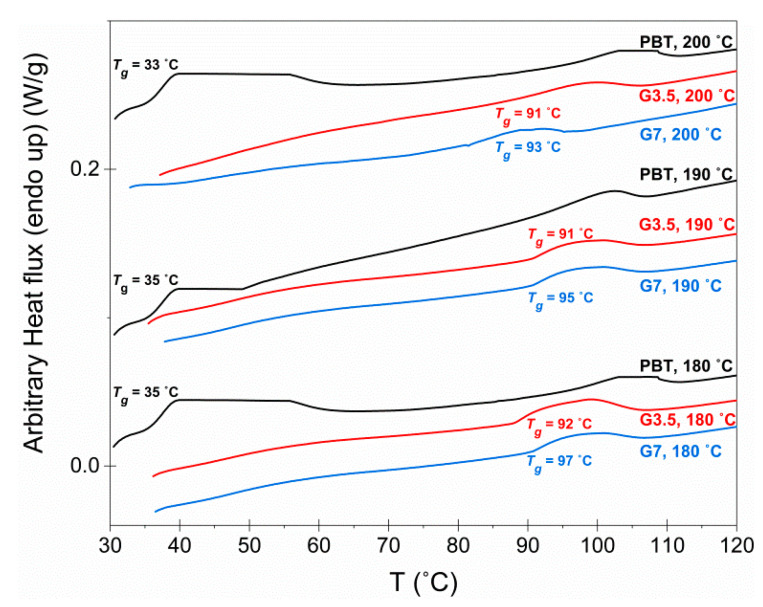
Differential scanning calorimetry (DSC) curves of the neat PBT powder and vitrimers at the end of the solid-state repolymerization (*t* = 3 h) at three different temperatures (180, 190, 200 °C).

In addition, based on the DSC curves (Figure A1), *T_g_* transition became broader in vitrimers, probably due to the random distribution of the dynamic crosslinks in the amorphous phase. However, the amount of glycerol was not found to play a significant role in the crosslink density since G3.5 and G7 vitrimers exhibited similar *T_g_*, with G7 presenting slightly higher values. This implies that the crosslinker amount notably affects the rate of crosslinking reactions based on *G_f_*, but the crosslink density is essentially governed by the selected SSP temperature: for both grades, *T_g_*_1_ was the highest at the lowest *T_SSP_* = 180 °C compared to the other two *T_SSP_*, meaning a denser chemical network. Regarding melting, *T_m_*_1_ increased up to 216 °C and 214 °C in PBT vitrimers and *x_c_*_1_ reached 33.2% and 34.6% for G3.5 and G7, respectively (Table 3), probably ascribed to the lamellar thickening that occurred simultaneously during SSP. However, this *T_m_*, which corresponds to the crystalline portion of the vitrimer material that did not participate in the transesterification reactions, was about 10 °C lower in contrast to neat pulverized PBT and obviously attributed to the loss of symmetry perfection under the influence of the crosslinks. 

In order to erase the effect of thermal annealing and juxtapose the thermal properties purely as a function of glycerol content and *T_SSP_*, the data from the second heating cycle were also analyzed. Overall, the same trends as in the first heating cycle were observed, practically showing that PBT molecule modification was indeed attained with the SSP step, yet the values were slightly decreased: the melt crystallization during cooling resulted in a different crystalline morphology with more defects. Similar results were found in another study on PBT-based vitrimers thermal properties [13,14], where *T_m_*_2_ was found 212 °C (indicatively for a sample with 7 mol % glycerol, produced at 180 °C for 3 h), corresponding with our results (*T_m_*_2_ = 210 °C for G7 produced at 180 °C, Table 3). Finally, decomposition of all vitrimers took place in one step at higher temperatures with respect to neat pulverized PBT, i.e., *T_d,_*_5*%*_ ranging from 363 to 371 °C and *T_d_* from 405 to 411 °C (Table 3).

During SSP, the transesterification reactions that occurred in the PBT’s amorphous regions (Figure 1b) altered the materials morphology: *a_rigid_* was increased, and *a_mobile_* was reduced for both G3.5 and G7 vitrimers, while *x_c_* reached the values of post-SSP, pure PBT. The mobile amorphous regions decreased due to the limited chain mobility, which was caused by the network development, especially for the case of G7 (3 h at 180 °C) in which *a_mobile_* reduced from 29.0% (Table 2) to 16.3% (Table 3). Therefore, *a_mobile_* possibly transformed to either *a_rigid_* or *x_c_* by lamellar thickening that occurred simultaneously during SSP, since *x_c_* was also increased for every vitrimer compared to each alcoholyzed product, as stated before. Zhou et al. [13] reported two divergent processes, during SSP, before and after the gel point: up to the gel point (*t_ssp_* < 3 h), *a_rigid_* was transformed both into *a_mobile_* and crystal phase by crystal perfection, which in the current work was observed only during the alcoholysis step. After the gel point (*t_ssp_* > 3 h), *a_mobile_* decreased from about 35% to 28%, and *a_rigid_* increased from about 13% up to 20%, concurrent with our reported trends.

**Non-isothermal melt crystallization study of PBT vitrimers**

**Figure 3:**
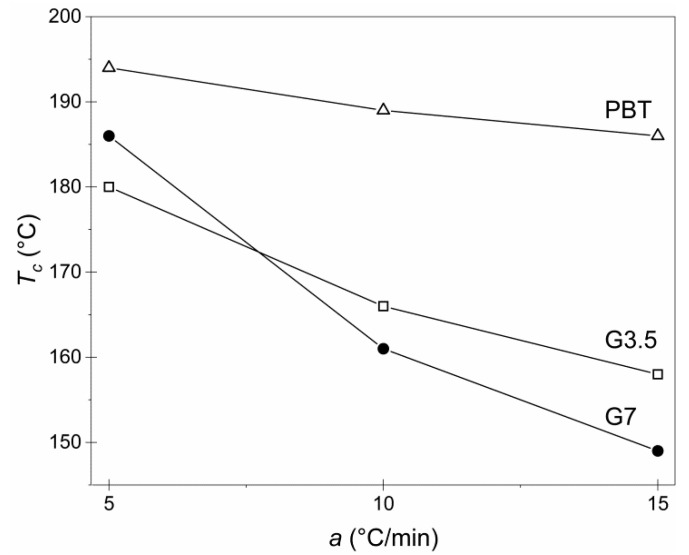
*T_c_* at each cooling rate of neat PBT and vitrimers G3.5 and G7 all three produced at the optimum SSP conditions (180 °C, 3 h).

Despite the recent exciting advances on vitrimers synthesis, no study has been done up to now on their melt crystallization behavior. Poor control of crystallization during the subsequent (re)processing may lead to widely varying mechanical properties and dimensional variations. For example, an injection molding plastic that is partially crystallized or forms a thermodynamically unstable intermediate may undergo further crystallization in secondary processing, storage and transport or even end-use, altering the physical properties of the material [16,24,26]. Therefore, it is essential to investigate the potential influence of the dynamic crosslinked network upon the crystallization behavior and, consequently, the final properties of PBT-based vitrimers. In this perspective, the neat PBT and the vitrimers G3.5 and G7 submitted at the optimum SSP temperature (180 °C) were studied regarding the non-isothermal melt crystallization. Data for the crystallization temperature *T_c_* as a function of the cooling rate *a* (5 °C/min, 10 °C/min and 15 °C/min) were obtained and depicted in Figure 3.

The materials were still able to crystallize, a phenomenon that further validates the dynamic nature of the developed crosslinking for both G3.5 and G7 vitrimers: PBT crystalline segments were largely maintained after the solid state repolymerization, as they did not participate in the transesterification reactions, and enabled the melt crystallization. Starting with the effect of cooling rate, as anticipated, the highest *T_c_* was achieved at the lowest cooling rate (5 °C/min) since the polymer chains had sufficient time to crystallize and the crystallization was nucleation-controlled. At each cooling rate, vitrimers presented lower *T_c_* values compared to neat PBT powder due to the crosslinked structure, which impeded crystallization. When comparing G3.5 and G7, at the cooling rates of 10 °C/min and 15 °C/min, the G7 vitrimer exhibited lower *T_c_*, meaning that the crystallization process initiated and concluded later due to the slightly denser crosslinked network. This resulted in the generation of crystal imperfections, which enhanced the crosslinking between the polymer and the glycerol, reduced their intermolecular mobility and ability to further crystallize, finally constraining the ultimate crystallinity.

**Figure 4:**
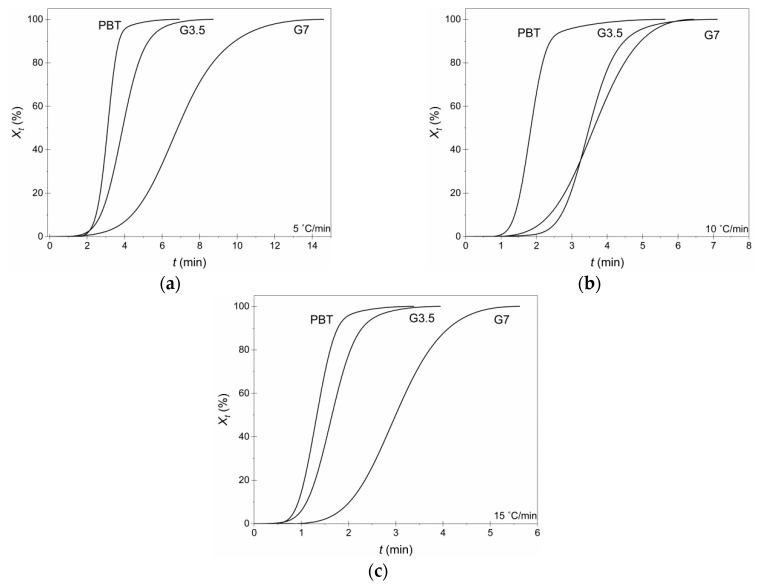
*X_t_* vs. *t* for pure PBT, G3.5 and G7 vitrimers (3 h at 180 °C) at each cooling rate: (**a**) 5 °C/min, (**b**) 10 °C/min and (**c**) 15 °C/min.

Regarding crystallization kinetics, Figure 4 shows the relative crystallinity *X_t_*; no changes were observed in the crystallization mechanism (primary crystallization) since conversion curves were of a similar type, shifted, however, to higher crystallization times in the case of vitrimers. It is safe to imply that the radial growth of crystals occurs at a constant velocity, and the impingement of crystals with one another was deemed negligible [30], and hence, the crystallization evolution can be quantitatively analyzed using the Avrami model [27,28]. *X_t_*_,_ as a function of time, could be calculated from Equation (10) [27,28]:

(10) $$X_{t}=1-\exp\left(-Z_{t}\;\cdot \;\;t^{n}\right)\underset{\;}{\Leftrightarrow }log\left(-ln\left(1-X_{t}\right)\right)=logZ_{t}+n\;\cdot \;logt$$

where *n* is the Avrami exponent, which denotes the nucleation process and geometry of crystallization, and *Z_t_* is the crystallization rate constant, which depends upon the nucleation and the growth of crystals.

The units of the *Z_t_* are a function of *n* and the constant temperature change during the melt crystallization, and hence, Equation (10) needed to be modified to study the non-isothermal crystallization kinetics as proposed by Jeziorny [31] by using the crystallization constant *K_A_* (s^−1^ or min^−1^), where ${Ζ}_{t}={\left(K_{A}\right)}^{n}$, into Equation (11):

(11) $$log\left(-ln\left(1-X_{t}\right)\right)=n\;\cdot \;logK_{A}+n\;\cdot \;logt$$

**Figure 5:**
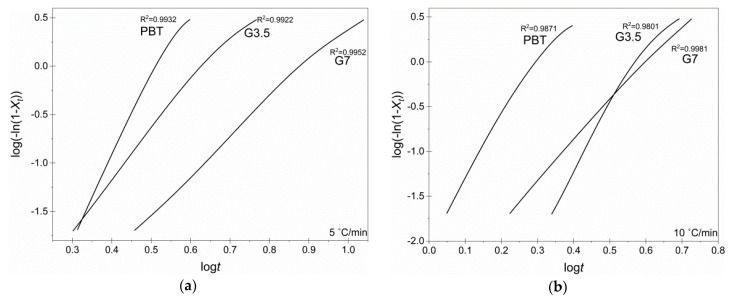

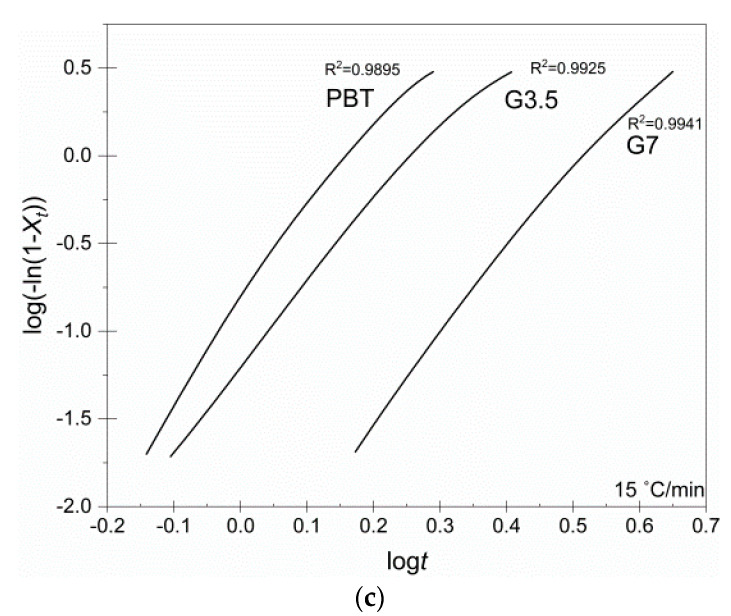
Avrami-type plots for pure PBT, G3.5 and G7 vitrimers (3 h at 180 °C with *X_t_* ranging from 2 to 95%, at each cooling rate: (**a**) 5 °C/min, (**b**) 10 °C/min and (**c**) 15 °C/min.

In order to fit the experimental results into the Avrami model, linear plots of the standard $log\left(-ln\left(1-X_{t}\right)\right)$ versus logt were constructed for the regime with *X_t_* between 2 and 95% (Figure 5). The calculated half time of crystallization *t_1/2_* (*Χ_t_* = 50%), *n* and *K_A_* for each material, as a function of *a* are summarized in Table 4. 

The *t*_1/2_ is a measure of the overall crystallization rate, and it was increasing in the vitrimers compared to neat PBT, proving that the crosslinks reduced crystallization rate, an observation more evident with higher glycerol concentration. Additionally, *n* is known to be affected by the molecular weight, nucleation type but, in general, not much influenced by the crystallization temperature [28]. Ideally, *n* should be an integer, as it strongly depends on both the crystals morphology and nucleation mechanism [32,33]. Herein, *n* for neat PBT ranged from 2.7 to 3, implying a diffusion-limited self-nucleation by 3-D spherulitic growth geometry, which is in perfect alignment with previously reported studies for pristine PBT [30,34,35]. The *n* values for the vitrimers were found increased in the vicinity of 4, implying an alteration in the crystallization mechanism towards more sporadic heterogeneous 3-dimensional spherulitic growth; the *n* increase was more intense (ca., *n* = 4.5) for G7 caused by the denser crosslinked network and increased rigidity of vitrimer. Finally, the pulverized PBT demonstrated the higher *K_A_* at every cooling rate compared to the produced vitrimers.

The Kissinger model (Equation (12)) was further used to assess the effective activation energy *Ε*_a_ for the melt crystallization [36]. More specifically, $ln\left(a/T_{c}{}^{2}\right)\;$*versus* $1/T_{c}$ plots were constructed, in which the slope corresponded to −*Ε_a_/R*:

(12) $$ln\left(\frac{a}{T_{c}{}^{2}}\right)=ln\left(\frac{A\;\cdot \;R}{E_{a}}\right)-\frac{E_{a}}{R\;\cdot \;T_{c}}\;$$

where *E_a_* (kJ/mol) is the overall effective activation energy, *R* (J∙mol^−1^∙K^−1^) is the gas constant equal to 8.314 J∙mol^−1^∙K^−1^, and *A* (min^−1^) is the pre-exponential factor.

**Figure 6:**
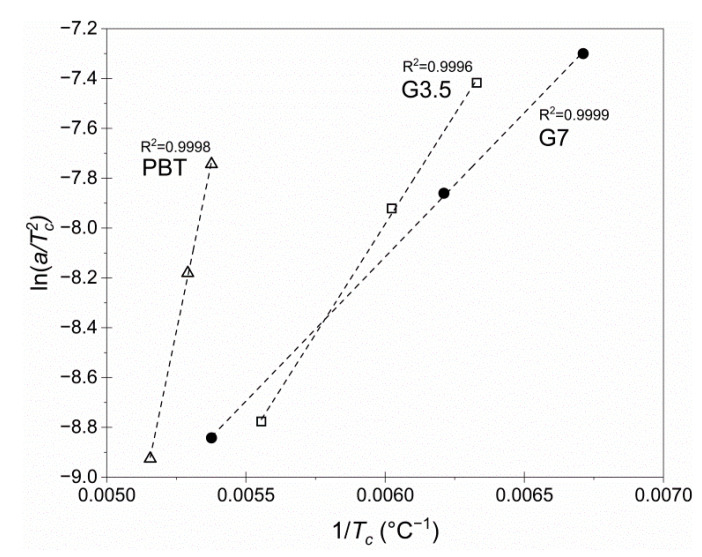
Arrhenius plots for the neat PBT, G3.5 and G7 vitrimers at the optimum SSP conditions (3 h at 180 °C).

The plots were found linear with excellent fitting (*R*^2^ > 0.9996), as shown in Figure 6. Interestingly, the calculated overall effective activation energies of vitrimers G3.5 (14.7 kJ/mol) and G7 (9.6 kJ/mol) were decreased, compared to the pure powder PBT (44.8 kJ/mol) (Table 4). Generally, *E_a_* of the overall crystallization can be ascribed to two types of activation energies, one for nucleation and one for growth, which are considered to be condensed into a single energy barrier since they are separated by very low values. Nucleation is the primary step, generally governed by nucleating agents, whereas the subsequent crystal growth can be impeded by crosslinks [37]. Herein, glycerol moieties and possibly the catalyst topologically acted as nucleation sites in the crosslinked structure and enabled nucleation, which progressively reduced the energy required for nucleation and thus, *E_a_* overall decreased. However, this *E_a_* reduction does not mean a faster crystallization, as the limited chain mobility due to permanent crosslinking hindered the subsequent growth of crystalline regions, leading to a slower crystallization overall for the vitrimers as previously discussed. The same trend has also been reported by Jose et al. [37], in which the reduction of the overall *E_a_* values of crosslinked polyethylene (XLPE) was ascribed to easier nucleation under the influence of a nucleating agent (ZnO), from 265 to 253 kJ/mol with increasing ZnO content from 2 to 10 wt. %. Finally, the lower *E_a_* results in vitrimers suggested that crystallization as a process was less affected by temperature compared to pure PBT.

In order to investigate the nucleating effect that glycerol moieties exhibited, the nucleating activity of foreign substrates in the polymer melt was also investigated, using the well-established method suggested by Dobreva and Gutzow [38,39]. Nucleating activity *φ* is the factor by which the work of three-dimensional nucleation decreases with the addition of foreign substrates, such as glycerol moieties in the crosslinked structure. If the foreign substrate is extremely active, *φ* reaches zero, while for dormant particles, *φ* approaches one. The nucleating activity can be calculated using Equation (13):

(13) $$\varphi =\frac{Β}{{Β}^{*}}\;$$

where *B* corresponds to homogenous nucleation (pure PBT) and B* to heterogeneous nucleation (G3.5 and G7). In particular, *B* is a parameter that can be computed using the Equation (14):

(14) $$B=\frac{\omega \;\cdot \;\sigma^{3}\;\cdot \;V_{m}{}^{2}}{3\;\cdot \;n\;\cdot \;k_{B}\;\cdot \;T_{m}^{{}^{\circ}}\;\cdot \;\Delta S_{m}{}^{2}}$$

where *ω* is a geometric factor, *σ* is the specific energy, *V_m_* (L/mol) is the molar volume of the crystallizing substance, *n* is the Avrami exponent, Δ*S_m_* (J/K) is the entropy of melting, and $T_{m}^{{}^{\circ}}$ (°C) is the infinite crystal melting temperature.

However, *B* can be easily determined experimentally from the standard plots of lna versus $1/\Delta {Τ}_{c}{}^{2}$, in which $\Delta {Τ}_{c}=T_{m}-T_{c}$ and the slope corresponds to −*B*, as suggested by Equation (15):

(15) $$lna=Constant-\frac{B\;or\;B^{*}\;}{\Delta T_{c}{}^{2}}\;$$

**Figure 7:**
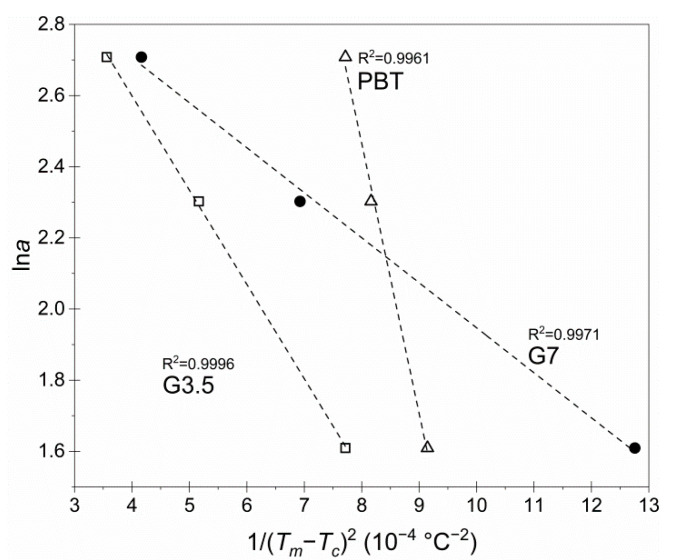
Plots of lna versus $1/\Delta {Τ}_{c}{}^{2}$ for the neat PBT, G3.5 and G7 vitrimers at the optimum SSP conditions (3 h at 180 °C).

Figure 7 demonstrates the standard plots of lna versus $1/\Delta {Τ}_{c}{}^{2}$ for pure PBT and vitrimers G3.5 and G7, in which straight lines were obtained in every sample (*R*^2^ > 0.9960). From the slopes of these lines, the values of *B* and B^*^ were calculated.

Subsequently, the values of *φ* for each material were computed using Equation (13), and the experimental results are demonstrated in Table 4. The values of *φ* revealed that both the G3.5 and G7 vitrimers exhibited some nucleation effect (*φ* < 1) since the glycerol-enriched developed crosslinks topologically acted as nucleation agents and enabled nucleation, which further validates the previously reported *E*_a_ reduction. Moreover, G7 vitrimer exhibited an even lower *φ* value (0.167) compared to G3.5 (0.349), due to the increased concentration of glycerol moieties in the macromolecular structure. Interestingly, the dense, dynamic crosslinking development had contrasting consequences overall: on the one hand, chain imperfections and glycerol-enriched microdomains had a nucleation effect which lowered the energy barrier for crystallization, whereas, on the other hand, permanent crosslinks reduced the crystallizability and the overall crystallization rate. At a low glycerol concentration (vitrimer G3.5), these effects basically counterbalance each other, while at a high glycerol concentration (vitrimer G7), crystal growth is even more limited and hence, crystallization overall was impeded.

## 4. Conclusions

The present work investigated the optimization of PBT-based vitrimers production via a two-step SSP process. SSP was found to efficiently develop vitrimers with a characteristic reversible dynamic network responsible for their ability to reprocess/recycle under heating while maintaining their crosslinking integrity and insolubility. The optimum *T_SSP_* was 180 °C, in which the highest crosslinking extent (*G_f_* = 75%) and highest melt strength (MFR = 13.8 g/10 min) were achieved. Moreover, by increasing the crosslinker content (from 3.5 to 7 wt. %), advanced properties were obtained in general, whereas at elevated SSP temperatures (190 and 200 °C), the thermal decomposition of the polymer and the catalyst was accelerated, leading to inferior final properties. DSC revealed that the semi-crystalline character of PBT was maintained since vitrimers demonstrated *x_c_* (i.e., 34.6% for G7) similar to post-SSP pure PBT (34.2%). Therefore, it can be assumed that the crosslink points were exclusively located in the amorphous phase, which almost tripled *T_g_* (up to 97 °C for G7). Critically, all end products displayed increased thermal stability (*T_d_*, *T_d,_*_5*%*_) with only moderately reduced *T_m_* compared to pure PBT, caused by crystal imperfections under the influence of the crosslinks. Regarding melt crystallization, the materials were still able to crystallize, which further validated the dynamic nature of the developed crosslinking. The dense macromolecular architecture hindered the crystallization process in general (lower *T_c_*, *K_A_* and *t*_1/2_ values) and resulted in an alteration of the crystallization mechanism towards more sporadic heterogeneous growth. Vitrimers exhibited lower *E*_a_ values compared to neat PBT, which implied that their crystallization was less affected by temperature. Interestingly, glycerol moieties in the crosslinked structure had a nucleation effect, which lowered the energy barrier for crystallization, but on the other hand, they reduced the crystallizability and the overall crystallization rate. Taking into account the above, it can be concluded that SSP comprises an efficient and promising path for the production of thermoplastic-based vitrimers with tailor-made attributes, and further work needs to be done so as to extend their potential applications as engineering polymers.

## Figures and Tables

**Table 1:** Intrinsic viscosity [η], $\bar{M_{n}}$ and melt flow rate (MFR) of the neat poly(butylene terephthalate) (PBT) powder, the physical mixtures (G3.5, G7) and the relevant alcoholysis products (*t* = 24 h, 160 °C).

Sample	[*η*] (dL/g)	$\bar{M_{n}}$ (g/mol)	MFR (g/10 min)
neat PBT powder (*t* = 0)	0.86 ± 0.06	29,000	29.3 ± 0.3
neat PBT powder (*t* = 24 h, 160 °C)	0.83 ± 0.05	28,000	32.1 ± 0.4
G3.5 (*t* = 0)	0.93 ± 0.03	32,000	24.2 ± 0.6
G3.5 (*t* = 24 h, 160 °C)	0.38 ± 0.03	13,000	59.6 ± 0.7
G7 (*t* = 0)	1.01 ± 0.07	35,000	21.7 ± 0.5
G7 (*t* = 24 h, 160 °C)	0.45 ± 0.01	10,000	66.0 ± 0.5

**Table 2:** Thermal properties of the neat PBT powder, physical mixtures (G3.5, G7) and the relevant alcoholysis products (*t* = 24 h, 160 °C).

Sample	DSC								TGA		
	1st Heating					2nd Heating					
	*T_g_*_1_ (°C)	*T_m_*_1_ (°C)	*x_c_*_1_ (%)	*a_mobile_* (%)	*a_rigid_* (%)	*T_g_*_2_ (°C)	*T_m_*_2_ (°C)	*x_c_*_2_ (%)	*T_d_* (°C)	*T_d,_*_5*%*_ (°C)	Residue (%)
Neat PBT powder (*t* = 0)	35	230	35.3	21.1	43.6	35	230	34.2	391	361	6.3
Neat PBT powder (*t* = 24 h, 160 °C)	33	225	33.7	23.5	42.8	31	224	30.2	391	361	6.4
G3.5 (*t* = 0)	84	222	32.2	10.8	57.0	83	213	31.7	398	360	7.9
G3.5 (*t* = 24 h, 160 °C)	50	208	28.7	29.0	42.3	51	199	25.6	398	358	8.1
G7 (*t* = 0)	89	214	30.6	6.6	62.8	89	206	27.9	398	361	8.1
G7 (*t* = 24 h, 160 °C)	46	192	23.5	55.9	20.6	46	186	22.7	398	357	8.0

**Table 3:** Thermal properties of the neat PBT powder and vitrimers at the end of the solid-state repolymerization (t = 3 h) at three different temperatures (180, 190, 200 °C).

Sample	DSC								TGA		
	1st Heating					2nd Heating					
	*T_g_*_1_ (°C)	*T_m_*_1_ (°C)	*x_c_*_1_ (%)	*a_mobile_* (%)	*a_rigid_* (%)	*T_g_*_2_ (°C)	*T_m_*_2_ (°C)	*x_c_*_2_ (%)	*T_d_* (°C)	*T_d,_*_5*%*_ (°C)	Residue (%)
Neat PBT powder (180 °C)	35	230	33.9	20.1	46.0	32	227	31.0	401	361	5.9
Neat PBT powder (190 °C)	35	225	34.2	23.5	42.3	33	224	30.9	401	361	5.9
Neat PBT powder (200 °C)	33	225	33.6	23.5	42.9	33	223	31.1	400	361	6.0
G3.5 (180 °C)	92	216	33.2	13.5	53.3	90	211	32.0	406	367	8.0
G3.5 (190 °C)	91	212	32.5	15.9	51.6	90	210	29.8	408	363	8.0
G3.5 (200 °C)	91	211	31.6	17.1	51.3	87	210	27.9	405	364	8.0
G7 (180 °C)	97	214	34.6	16.3	49.1	96	210	33.8	411	371	8.1
G7 (190 °C)	95	201	33.4	20.7	45.9	93	196	28.9	407	368	7.9
G7 (200 °C)	93	198	33.2	22.2	44.6	90	195	28.0	405	368	8.0

**Table 4:** Crystallization parameters for the neat PBT, G3.5 and G7 vitrimers at the optimum SSP conditions (3 h at 180 °C).

Sample	Cooling Rate a (°C/min)	*t*_1/2_ (min)	*n*	*K_A_* (min^−1^)	*E_A_* (kJ/mol)	Nucleating Activity *φ*
Neat PBT powder	5	3.08	2.69	0.0319	44.8	1.000
Neat PBT powder	10	1.86	2.86	0.2274		
Neat PBT powder	15	1.33	2.99	0.5255		
G3.5	5	3.91	3.78	0.1557	14.7	0.349
G3.5	10	3.49	4.27	0.1765		
G3.5	15	1.65	4.37	0.5396		
G7	5	6.83	4.16	0.1495	9.6	0.167
G7	10	3.63	4.30	0.2495		
G7	15	2.99	4.49	0.2996		

## Data Availability

The data presented in this study are available on request from the corresponding author. The data are not publicly available due to at this time due to technical or time limitations.
